# The Long-Term Effectiveness of Internet-Based Interventions on Multiple Health Risk Behaviors: Systematic Review and Robust Variance Estimation Meta-analysis

**DOI:** 10.2196/23513

**Published:** 2021-12-21

**Authors:** Flora Tzelepis, Aimee Mitchell, Louise Wilson, Emma Byrnes, Alexandra Haschek, Lucy Leigh, Christopher Oldmeadow

**Affiliations:** 1 School of Medicine and Public Health University of Newcastle Callaghan Australia; 2 Hunter New England Population Health Hunter New England Local Health District Wallsend Australia; 3 Hunter Medical Research Institute New Lambton Heights Australia; 4 School of Psychology and Public Health La Trobe University Bundoora Australia; 5 The Cooperative Research Centre for Living with Autism Brisbane Australia

**Keywords:** internet, multiple health behaviors, tobacco, nutrition, alcohol, physical activity

## Abstract

**Background:**

Smoking tobacco, poor nutrition, risky alcohol use, and physical inactivity (SNAP) behaviors tend to cluster together. Health benefits may be maximized if interventions targeted multiple health risk behaviors together rather than addressing single behaviors. The internet has wide reach and is a sustainable mode for delivery of interventions for multiple health behaviors. However, no systematic reviews have examined the long-term effectiveness of internet-based interventions on any combination of or all SNAP behaviors in adults aged 18 years or older.

**Objective:**

This systematic review examined, among adults (aged ≥18 years), the effectiveness of internet-based interventions on SNAP behaviors collectively in the long term compared with a control condition.

**Methods:**

The electronic databases Medline, PsycINFO, Embase, CINAHL, and Scopus were searched to retrieve studies describing the effectiveness of internet-based interventions on ≥2 SNAP behaviors published by November 18, 2019. The reference lists of retrieved articles were also checked to identify eligible publications. The inclusion criteria were randomized controlled trials or cluster randomized controlled trials with adults examining an internet-based intervention measuring the effect on ≥2 SNAP behaviors at least 6 months postrecruitment and published in English in a peer-reviewed journal. Two reviewers independently extracted data from included studies and assessed methodological quality using the Quality Assessment Tool for Quantitative Studies. A robust variance estimation meta-analysis was performed to examine the long-term effectiveness of internet-based interventions on all 4 SNAP risk behavior outcomes. All SNAP outcomes were coded so they were in the same direction, with higher scores equating to worse health risk behaviors.

**Results:**

The inclusion criteria were met by 11 studies: 7 studies measured the effect of an internet-based intervention on nutrition and physical activity; 1 study measured the effect on smoking, nutrition, and physical activity; and 3 studies measured the effect on all SNAP behaviors. Compared with the control group, internet-based interventions achieved an overall significant improvement across all SNAP behaviors in the long term (standardized mean difference –0.12 [improvement as higher scores = worse health risk outcomes], 95% CI –0.19 to –0.05; I^2^=1.5%, *P*=.01). The global methodological quality rating was “moderate” for 1 study, while the remaining 10 studies were rated as “weak.”

**Conclusions:**

Internet-based interventions were found to produce an overall significant improvement across all SNAP behaviors collectively in the long term. Internet-based interventions targeting multiple SNAP behaviors have the potential to maximize long-term improvements to preventive health outcomes.

## Introduction

Smoking tobacco, poor nutrition, risky alcohol use, and physical inactivity (SNAP) are modifiable risk factors for chronic diseases such as heart disease, stroke, cancer, and diabetes [1]. Individuals who engage in all 4 SNAP behaviors, compared with 0, have an increased risk of mortality, equivalent to 14 years of aging [2]. Evidence has shown that SNAP behaviors tend to cluster together [3-5], suggesting a holistic approach for interventions to modify multiple health behaviors collectively rather than single behaviors individually may be beneficial. Multiple health behavior interventions target 2 or more health behaviors either sequentially or simultaneously [6]. Advantages of improving multiple health behaviors include maximizing health benefits [7], greater reduction in medical costs [8], and successfully modifying one behavior may increase confidence or motivation to change other health behaviors [7,9].

The internet is accessible globally and is a sustainable mode for the delivery of interventions for multiple health behaviors [10]. There are more than 4 billion internet users worldwide [10]; therefore, internet-based interventions have the potential to reach large numbers of people. Other advantages of internet-based interventions include that users can access information any time [11,12] as well as a low-cost modality for information delivery [11,12] and allowing for privacy, confidentiality [11], and long-term use [12,13]. Internet-based interventions may be interactive [11-13] and incorporate behavior change techniques such as individually tailored information [11,13], goal setting [12-14], self-monitoring [12-14], personalized and normative feedback [11,12,14], and progress tracking [12,13]. Internet-based interventions may also reduce health inequalities by improving access to services, for example among individuals who live in rural and remote areas or have significant mobility issues [13,15].

Existing systematic reviews have examined the effectiveness of behavioral interventions on multiple health risk behaviors [16,17]. A systematic review of nonpharmacologic interventions on multiple health risk behaviors found modest improvements in fruit and vegetable intake, physical activity, reduced fat intake, and reduced smoking [16]. Furthermore, another systematic review examining the efficacy of apps in children, adolescents, and adults reported that 41% of multiple health behavior interventions showed significant between-group improvements in behaviors [17]. However, only 2 systematic reviews have specifically examined the effectiveness of internet-based interventions on 2 or more SNAP behaviors in adult populations [18,19]. The review by Norman et al [18] focused on interventions for nutrition and physical activity but not tobacco use and alcohol intake. Of the 17 studies targeting multiple behaviors, 6 studies favored an internet-based intervention for increasing physical activity, and 6 studies favored an internet-based intervention for changing nutrition behaviors [18]. However, this systematic review was not restricted to adult populations, and findings from children and adolescents were included in the synthesis of findings [18]. Furthermore, short-term follow-up assessments were contained within this systematic review, and many studies did not report the effect of the internet-based intervention on nutrition and physical activity in the long term [18]. In the second systematic review, Oosterveen and colleagues [19] examined the effectiveness of internet-based interventions on combinations of all SNAP behaviors but included young adults aged 18 years to 35 years only. This systematic review identified only 2 studies with young adults targeting nutrition and physical activity behaviors that included a long-term follow-up (ie, 6 months or longer) [19]. To our knowledge, there is no systematic review that has examined the long-term effectiveness of internet-based interventions on any combination of or all SNAP behaviors in adults aged 18 years or older. Further critical review of the evidence is therefore needed to understand whether internet-based interventions are effective in improving multiple SNAP behaviors in the long term.

This systematic review aimed to examine the effectiveness of internet-based interventions on multiple SNAP health risk behaviors in the long term compared with a control condition.

## Methods

### Search Strategy and Selection Criteria

The electronic databases Medline, PsycINFO, Embase, CINAHL, and Scopus were searched to retrieve studies describing the effectiveness of internet-based interventions on 2 or more SNAP behaviors published by November 18, 2019. The following combinations of keywords were used: (multiple health behavio* or multiple behavio* or multiple risk* or multiple health* or smok* or tobacco or alcohol or diet* or nutrition or exercise or physical activity or fruit* or vegetable*) AND (internet or web* or online or on-line) AND (trial* or RCT* or random*). The reference lists of retrieved articles were also checked to identify any additional eligible publications.

The inclusion criteria were studies (1) that reported randomized controlled trials (RCTs) or cluster RCTs of internet-based interventions for ≥2 SNAP behaviors as either the sole intervention or an adjunct to written materials, (2) with adults aged 18 years or older, (3) that reported outcomes for ≥2 SNAP behaviors at least 6 months postrecruitment, (4) that had a no-intervention control group or the control group received information either in hard copy or information unrelated to SNAP via a website, and (5) in the English language in a peer-reviewed journal.

Publications were excluded if (1) they did not report the outcomes of an RCT or cluster RCT (eg, systematic reviews, commentaries); (2) they examined only 1 SNAP health behavior; (3) they included special populations only such as people with chronic conditions (eg, cancer, diabetes) or pregnant women (this criterion was chosen because people with chronic conditions may differ in their motivation and capacity to change behaviors compared with those without chronic conditions and is consistent with the criterion set in another systematic review that examined multiple health risk behaviors [16]); (4) were conducted with people under 18 years of age; (5) outcome measures were not related to SNAP (eg, blood pressure); (6) the internet-based intervention was part of a multicomponent approach that included other modes of support (eg, face-to-face, telephone); (7) there was no control arm, and instead, comparisons were made with other interventions (eg, face-to-face support); and (8) SNAP outcomes were measured before 6 months postrecruitment.

### Selection of Eligible Studies

This systematic review was conducted in accordance with PRISMA (Preferred Reporting Items for Systematic Reviews and Meta-Analyses) guidelines [20]. All records identified in each electronic database were imported into Endnote, and duplicates were removed. Titles, abstracts, and full texts of each reference were independently screened in duplicate by 2 reviewers (LW and FT or AM or EB) to determine if eligibility criteria were met. Full-text articles were retrieved when eligibility could not be determined from the title and abstract screening.

### Study and Sample Characteristics

Data were independently extracted from the included studies by 2 authors (AM and AH or LW). A third author (FT) resolved any inconsistencies in data extraction. The study and sample characteristics extracted from eligible publications included authors and year of publication, country, years that data were collected, setting, sample characteristics (eg, mean age, gender, education, employment status), recruitment method, eligibility criteria, treatment conditions (relevant arms only; ie, internet-based intervention and control arms), internet-based intervention received (eg, duration, number of modules), retention rate at follow-up, SNAP measures, SNAP outcomes at 6 months of follow-up or later, and costs.

The outcomes extracted for each health behavior were any measure of (1) tobacco smoking (eg, current tobacco smoking, point prevalence abstinence, or prolonged abstinence), (2) nutrition (eg, number of daily serves of fruit and vegetables, dietary score), (3) alcohol consumption (eg, number of alcoholic drinks per day), and (4) physical activity (eg, moderate to vigorous physical activity, metabolic equivalent of task [MET] minutes per week).

### Methodological Quality Assessment

The Quality Assessment Tool for Quantitative Studies developed by the Effective Public Health Practice Project was used to assess methodological quality [21]. This tool was chosen because it has demonstrated content validity, construct validity, and test-retest reliability [22] and has been shown to have higher interrater reliability than the Cochrane Collaboration Risk of Bias Tool [23]. The Quality Assessment Tool for Quantitative Studies allows randomized trials to be rated on 6 components: (1) selection bias, (2) study design, (3) confounders, (4) blinding, (5) data collection methods, and (6) withdrawals and dropouts. Each study was rated as “strong,” “moderate,” or “weak” for each component. An overall global rating was then assigned to each study, with studies classified as “strong” (no weak ratings), “moderate” (1 weak rating), or “weak” (2 or more weak ratings). The Quality Assessment Tool for Quantitative Studies Dictionary was used to make judgments about each of the 6 components [24].

The methodological quality of included studies was rated by 2 authors (FT and AM). Any discrepancies were discussed between these authors until consensus was reached. When rating the data collection methods, the measures for “all” SNAP outcomes needed to be shown to be valid and reliable for the data collection methods to be rated as strong. For instance, if smoking cessation was measured via biochemical validation and physical activity assessed via pedometers, the data collection methods were rated as strong. However, if smoking cessation was measured via a self-reported measure with no information about its psychometric properties and pedometers were used to assess physical activity, the data collection methods were rated as weak because “all” SNAP measures were not shown to be reliable and valid.

### Robust Variance Estimation Meta-analysis

A robust variance estimation meta-analysis was performed using the R package *robumeta*. All SNAP behaviors were coded so they were in the same direction, with higher scores equating to worse health risk outcomes. Where a study measured the outcome at multiple time points (eg, 6 months and 12 months), data from the longer-term follow-up was included in the meta-analysis. The SNAP outcomes from each study were converted into Cohen d (standardized mean differences [SMDs]) and the corresponding variance [25]. Robust variance estimation meta-analysis was then performed on the SMDs (and variances), using the R package *robumeta*. A common within-study correlation (rho) of 0.8 was assumed, and sensitivity analyses were performed to determine the sensitivity of the results to this assumption (by looking at the results across various rho equal to 0, 0.2, 0.4, 0.6, or 1.0). Heterogeneity was measured using the I^2^ statistic [26].

## Results

### Search Results

**Figure 1:**
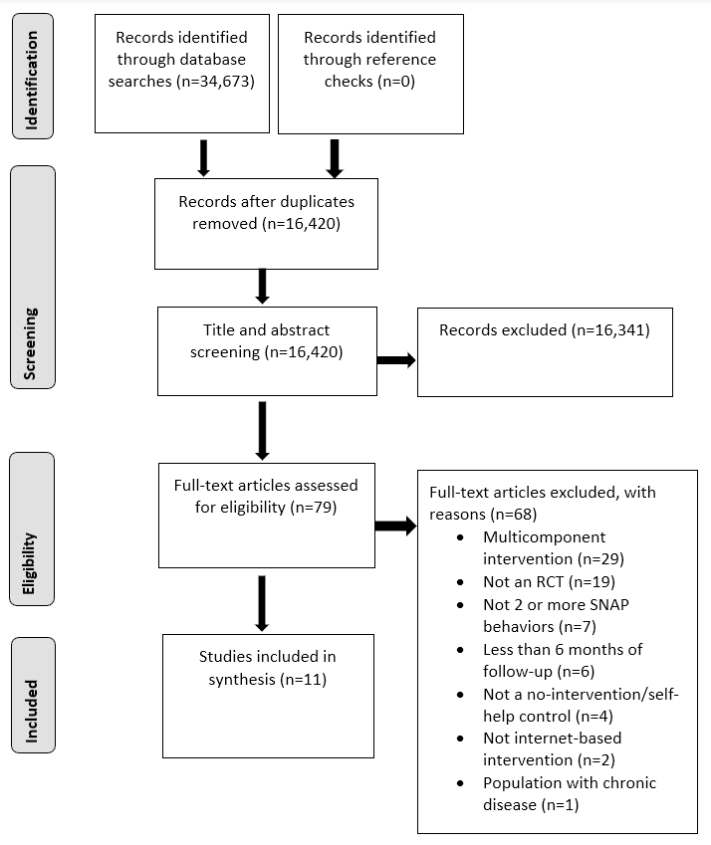
PRISMA (Preferred Reporting Items for Systematic Reviews and Meta-Analyses) diagram of the screening and selection process. RCT: randomized controlled trial.

The PRISMA flow diagram outlining the study selection process is presented in Figure 1. A total of 34,673 records were identified from the electronic database searches. After the removal of duplicates, 16,420 records had their title and/or abstract screened, and 16,341 did not meet the inclusion criteria. The full texts of the remaining 79 articles were retrieved for further review, and 68 articles were excluded for the following reasons: multicomponent intervention (n=29), not an RCT (n=19), did not measure 2 or more SNAP behaviors (n=7), less than 6 months of follow-up (n=6), did not include a no-intervention or self-help control group (n=4), not an internet-based intervention (n=2), and population with chronic disease (n=1). The inclusion criteria for this review were met by 11 studies.

### Study Characteristics of Internet-Based Interventions for Multiple Health Risk Behaviors

#### Studies That Examined Nutrition and Physical Activity

As shown in Multimedia Appendix 1, the effectiveness of an internet-based intervention on nutrition and physical activity was examined by 7 studies [27-33]; 5 studies were conducted in the United States [27,29,30,32,33], and 1 study each was conducted in the United Kingdom [31] and Australia [28]. Recruitment occurred from the community in 3 studies [28,32,33], from health care settings in 2 studies [27,31], and from universities in 2 studies [29,30]. In 6 studies, participants were recruited via various advertisements (eg, website, emails, newspaper, flyers, posters, radio) [27,28,30-33], while 2 studies used sign-up tables at universities [29,30]. The sample size ranged from 121 [27] to 1071 [33]. Only men were included in 2 studies [28,32], and only women were included in 1 study [27]. The percentage of women ranged from 56.3% (268/476) to 77% (171/221) in the remaining studies [29-31,33].

The interventions included web-based learning activities, modules, or tutorials [27,30,32,33]; self-monitoring [28,32]; educational materials [28,29,31]; social support [28]; tailored information [27,30-33]; feedback [32]; and goal setting [32,34]. The duration of engagement with the internet-based interventions ranged from 2 sessions [29] to a 12-month trial period [31]. Nutrition and physical activity were measured via self-reported measures in 6 studies [27-32], while 1 study used self-reported measures for nutrition and pedometers for physical activity [33]. Nutrition and physical activity were assessed at 6 months in 4 studies (range of retention of 291/441, 66.0% to 105/121, 86.8% [27,29,31,32]), 7 months in 1 study (retention: 950/1071, 88.7% [33]), 9 months in 1 study (retention: 148/317, 46.7% [28]), 12 months in 2 studies (retention: 131/221, 59.3% and 309/441, 70.1% [31,32]), 15 months in 1 study (retention: 1126/1689, 66.7% [30]), and 16 months in 1 study (retention: 935/1071, 87.3% [33]).

#### Studies That Examined Smoking, Nutrition, and Physical Activity

Multimedia Appendix 2 describes the study characteristics of the US study examining the effectiveness of an internet-based intervention on smoking, nutrition, and physical activity [34]. This study recruited 423 university staff (female: 347/423, 82%; mean age 51 years) via announcements on staff listservs, targeted emails, recruitment tables at events, and flyers [34]. The RealAge internet program generated individual risk profiles and allowed users to select behaviors to change and create plans to meet behavioral goals [34]. Self-reported measures assessed smoking cessation, nutrition, and physical activity at 6-month (retention: 360/423, 85.1%) and 12-month (retention: 367/423, 86.8%) follow-ups [34].

#### Studies That Examined Smoking, Nutrition, Alcohol, and Physical Activity

As outlined in Multimedia Appendix 3, the effectiveness of internet-based interventions on smoking, nutrition, alcohol, and physical activity was assessed by 3 studies [35-37]. All studies were conducted in the United Kingdom [35-37]; 2 studies were undertaken in the university setting [35,36], and 1 study was conducted in the community [37]. In 2 studies, incoming undergraduate students were recruited via an email invitation [35,36], while online and print advertisements were used to recruit participants in the community study [37]. Across the 3 studies, the sample size ranged from 100 [37] to 2621 [35]. Most participants were women in all the studies (range: 1447/2614, 55.4% to 82/100, 82% [35-37]), and the mean age ranged from 18.8 years [35] to 39 years [37]. The U@Uni [36] and U@Uni:LifeGuide [35] internet-based interventions included a profile page containing self-affirmation manipulation, theory-based messages for each SNAP behavior, and a planner to form implementation intentions. The HealthyValues Healthy Eating program targeted motivation, volition, and maintenance and included weekly tasks for 24 weeks [37]. All studies measured SNAP outcomes via self-reported measures at a 6-month follow-up [35-37]. The retention rates at 6 months were 41.2% (1079/2621) [35], 63.2% (913/1445) [36], and 95% (95/100) [37], respectively. 

### Long-Term Effectiveness of Internet-Based Interventions Across SNAP Risk Behaviors

The robust variance estimation meta-analysis found that, compared with the control group, internet-based interventions achieved an overall significant improvement across all SNAP behaviors in the long-term (SMD –0.12 [improvement as higher scores = worse health risk outcomes], 95% CI –0.19 to –0.05; I^2^=1.5%, *P*=.01) [27,29,30,32,34-37]. Heterogeneity was low (I^2^=1.5%), and the tau-square (the extent of variation due to between-study variance) was low, at <0.001. The sensitivity analysis showed that the model results did not vary greatly across different values of within-study correlation (rho).

### Methodological Quality Assessment

Table 1 presents the methodological quality ratings for the 6 components and the global rating as assessed via the Quality Assessment Tool for Quantitative Studies [21]. In terms of selection bias, all studies were rated “weak,” as 9 of these studies recruited volunteers who responded to advertisements or sign-up tables [27-34,37], and 2 studies sent emails to all incoming undergraduate students but recruited less than half of those approached [35,36]. All included studies were RCTs or cluster RCTs and were rated as “strong” in terms of study design [27-37]. With regards to confounders, 8 studies were rated as “strong” because there were no between-group differences at baseline [31,32,34,35,37] or adjustments for baseline characteristics were made during analysis [27,29,36], while 1 study was rated as “moderate,” as stratification attempted to balance baseline characteristics across groups [33], and 2 studies were rated as “weak,” as no adjustments were made during analysis for baseline characteristics that differed between the groups [28,30]. For blinding, 9 studies were rated as “weak” because the assessors and participants were not blinded [28,31,34] or there was no information about blinding [27,29,30,33,35,36], while 2 studies were rated as “moderate” because only the assessors were blinded to the condition [32,37]. For data collection methods, 8 studies were rated as “weak,” as all relevant SNAP measures were not shown to be valid or reliable (either via the use of an objective measure or demonstration of acceptable psychometric properties of a self-reported measure) [27,30,31,33-37], while 3 studies were classified as “strong,” as all SNAP measures used were valid and reliable [28,29,32]. In relation to withdrawals and dropouts, 4 studies were rated as “strong,” with retention rates ≥80% (105/121; 935/1071; 367/423; 95/100) [27,33,34,37]; 4 studies were rated as “moderate,” with retention rates between 60% and 79% (422/606; 1126/1689; 309/441; 913/1445) [29,30,32,36]; and 3 studies were rated as “weak,” with retention rates <60% (148/317; 131/221; 1079/2621) [28,31,35]. In terms of the global rating, 1 study was rated as “moderate” [32], and the remaining 10 studies were rated as “weak” [27-31,33-37].

**Table 1:** Methodological quality assessment of included studies.

Study		Selection bias	Study design	Confounders	Blinding	Data collection method	Withdrawals and dropouts	Global rating
**Nutrition and physical activity studies**								
	Drieling et al [27]	Weak	Strong	Strong	Weak	Weak	Strong	Weak
	Duncan et al [28]	Weak	Strong	Weak	Weak	Strong	Weak	Weak
	Franko et al [29]	Weak	Strong	Strong	Weak	Strong	Moderate	Weak
	Greene et al [30]	Weak	Strong	Weak	Weak	Weak	Moderate	Weak
	McConnon et al [31]	Weak	Strong	Strong	Weak	Weak	Weak	Weak
	Patrick et al [32]	Weak	Strong	Strong	Moderate	Strong	Moderate	Moderate
	Winett et al [33]	Weak	Strong	Moderate	Weak	Weak	Strong	Weak
**Smoking, nutrition, and physical activity study**								
	Hughes et al [34]	Weak	Strong	Strong	Weak	Weak	Strong	Weak
**Smoking, nutrition, alcohol, and physical activity studies**								
	Cameron et al [35]	Weak	Strong	Strong	Weak	Weak	Weak	Weak
	Epton et al [36]	Weak	Strong	Strong	Weak	Weak	Moderate	Weak
	Tapper et al [37]	Weak	Strong	Strong	Moderate	Weak	Strong	Weak

## Discussion

### Principal Findings

This is the first systematic review to examine the long-term effectiveness of internet-based interventions on SNAP behaviors collectively in adults aged 18 years or older. This systematic review focused on internet-based interventions to increase the homogeneity of included studies. This is similar to other systematic reviews that have focused on a specific digital technology [38,39]. More broadly, however, digital technologies can also include text messaging, email, mobile applications, video conferencing [40], and just-in-time feedback interventions [41]. The studies included in this systematic review most commonly examined effectiveness on 2 SNAP behaviors, namely nutrition and physical activity [27-33]. Only 3 studies examined the effectiveness of internet-based interventions on all 4 SNAP behaviors [35-37], whereas 1 study measured the effect on 3 behaviors (ie, tobacco smoking, nutrition, physical activity) [34].

The robust variance estimation meta-analysis findings reported that internet-based interventions achieved an overall significant improvement across all SNAP behaviors in the long term. This suggests that internet-based interventions that adopt a holistic approach to behavior change by addressing multiple SNAP behaviors improve these behaviors collectively and consequently may lead to better health outcomes and reduced health care costs. Given no previous systematic reviews have examined the long-term effectiveness of internet-based interventions on multiple SNAP behaviors nor provided an overall effect size across all SNAP behaviors, we cannot compare our findings to previous reviews. To advance the field, further research is needed on the long-term effectiveness of internet-based interventions on multiple SNAP behaviors.

The studies in this systematic review recruited participants from a variety of settings, including universities [29,30,34-36], the community [28,32,33,37], and health care [27,31]. Among the 5 studies conducted in universities, only 1 study reported a significant treatment effect on both nutrition and physical activity [30], while another study found the intervention reduced current smoking but had no effect on nutrition, alcohol, and physical activity [36]. Of 4 studies conducted in the community, 2 studies reported a significant treatment effect of the internet-based intervention on nutrition and some physical activity outcomes [32] or on some nutrition outcomes but not physical activity [33]. The trials that recruited participants from health care settings found no significant differences between the internet-based intervention and the control condition [27,31]. Additional research is needed across a variety of settings to expand the evidence base examining the long-term effectiveness of internet-based interventions on combinations of 2 or more SNAP behaviors.

In terms of methodological quality, 10 of 11 studies had a global rating of “weak” [27-31,33-37], with only 1 study rated as “moderate” [32]. Improvements to methodological rigor are particularly needed for selection bias, blinding, and data collection methods. Selection bias could be reduced by using recruitment methods that aim to enroll a representative sample (eg, random selection of potential participants) while data collection methods could be improved via objective measures (eg, pedometers for physical activity, biochemical validation for smoking cessation) for all SNAP behaviors assessed. Given the nature of behavioral interventions, blinding is often difficult; however, future studies should attempt to blind assessors and participants where possible.

### Limitations

This systematic review had some limitations. First, although we were able to pool the studies to undertake a robust variance estimation meta-analysis to examine the long-term effectiveness of internet-based interventions across all SNAP behaviors, additional analyses examining potential moderators (eg, country) were not possible due to the relatively small number of studies in this systematic review. Second, the methodological quality assessments were based on the information contained in the articles, and missing details from these articles may have had an impact on the ratings. Finally, all the studies were conducted in high-income countries, which may limit the generalizability of this systematic review’s findings to low- and middle-income countries. In addition to expanding the research in the settings and populations included in this review, future research should assess the long-term effectiveness of internet-based interventions on multiple SNAP behaviors in additional populations (eg, culturally and linguistically diverse groups, Indigenous), settings (eg, vocational education settings, rural and remote locations), and countries (eg, low- and middle-income) to strengthen the evidence base and improve the generalizability of the findings.

### Conclusions

Internet-based interventions were found to produce an overall significant improvement across all SNAP behaviors in the long term. Given the promising findings on the long-term effectiveness of internet-based interventions across all SNAP behaviors collectively, such interventions may maximize improvements to health and prevent chronic diseases.

## Appendix

Multimedia Appendix 1 Characteristics of studies examining the effectiveness of an internet-based intervention on nutrition and physical activity.

Multimedia Appendix 2 Characteristics of studies examining the effectiveness of an internet-based intervention on smoking, nutrition, and physical activity.

Multimedia Appendix 3 Characteristics of studies examining the effectiveness of an internet-based intervention on smoking, nutrition, alcohol, physical activity (SNAP).

## Abbreviations

MET: metabolic equivalent of task
NHMRC: National Health and Medical Research Council
PRISMA: Preferred Reporting Items for Systematic Reviews and Meta-Analyses
RCT: randomized controlled trial
SMD: standardized mean difference
SNAP: smoking, nutrition, alcohol, physical activity
