# Supplementary material for: The Long-Term Effectiveness of Internet-Based Interventions on Multiple Health Risk Behaviors: Systematic Review and Robust Variance Estimation Meta-analysis
Source: J Med Internet Res. 2021 Dec 21;23(12):e23513. doi: 10.2196/23513 (PMC8734928; doi:10.2196/23513)
Supplement: Multimedia Appendix 1 [file jmir_v23i12e23513_app1.docx]

**Multimedia Appendix 1: Characteristics of studies examining the effectiveness of an internet-based intervention on nutrition and physical activity**

| **Authors**  **Country**  **Years data collected** | | **Setting**  **Sample characteristics (sample size, demographics)** | **Recruitment method**  **Eligibility criteria** | **Treatment conditions (relevant arms)**  **Internet-based intervention received**  **Retention at follow-up** | **Measures of health behaviours** | **Health behaviours outcomes**  **Costs** |
| --- | --- | --- | --- | --- | --- | --- |
| Drieling et al [27]  USA  2008-2009 | Stanford in California  n=121  100% (121/121) women  69.4% (84/121) aged 51-70 years  86.0% (104/121) white  42.2% (51/121) postgraduate education  57.9% (70/121) employed  66.9% (81/121) married  18.2% (22/121) overweight, 11.6% (14/121) obese | | Ads at Stanford Hospital Clinics and on the Stanford Health Improvement Program website and e-mail announcement to prior visitors to Stanford Health Promotions Register website.  Female, aged 45-85 years, have internet access, willing to be randomised to intervention or control group, able to read English or have access to interpreter, no psychiatric disorder with active manifestations or substance abuse, do not live in the same residence as an enrolled participant, not a long-term resident of a nursing home, not homeless, no significant comorbidities | Internet (n=61): 18 tailored internet-based tutorials addressing modifiable factors (e.g. nutrition, exercise), unmodifiable factors, goal setting, behaviour change, and behavioural relapse prevention. 10 tutorials in first 3 months, 8 tutorials in final 3 months.  Control (n=60): Standard osteoporosis information from the National Osteoporosis Foundation website.  Average of 9.2 (SD=2.5) of 10 tutorials at 3 months and average of 6.1 (SD=3.2) of 8 tutorials at 6 months.  86.8% (105/121) retention at 6-months | Nutrition: Calcium and vitamin D intake via a brief food frequency questionnaire by the California Diary Council and dietary supplement questions by researchers.  Physical activity: Stanford Brief Physical Activity Survey and questions adapted from Centers for Disease Control Behavioral Risk Factor Surveillance System. | Nutrition outcomes  *Calcium intake (met recommendations)*  6-months: Internet: 72.1% (44/61); Control: 63.3% (38/60) (NS)  *Vitamin D intake* *(met recommendations)*  6-months: Internet: 65.6% (40/61); Control: 58.3% (35/60) (NS)  Physical activity outcomes  *Aerobic exercise (high/medium levels)*  6-months: Internet: 77.0% (47/61); Control: 73.3% (44/60) (NS)  *Resistance exercise (high/medium levels)*  6-months: Internet: 72.1% (44/61); Control: 66.7% (40/60) (NS)  Costs not stated |
| Duncan et al [28]  Australia  2010-2012 | | Gladstone and Rockhampton, Queensland  n=317  301 participants completed baseline survey  100% (301/301) men  Mean age: 44.17 years (Internet), 43.84 years (Control)  University education: 48.3% (99/205) (Internet), 53.1% (51/96) (Control)  Overweight: 41.5% (85/205) (Internet), 42.7% (41/96) (Control)  Obese: 49.3% (101/205) (Internet), 43.8% (42/96) (Control) | Local newspaper ads and trade magazine ads, face-to-face sessions with local business, leaflets and posters to local businesses, medical clinics and offices of allied health professionals.  Male, aged 35-54 years old, own a mobile phone, internet access, no mobility impairment, resided in Gladstone or Rockhampton (QLD, Australia) and classified as low risk to increase physical activity according to established guidelines. | Internet (n=214): Access to fully automated ‘ManUp’ website or mobile web application to self-monitor PA and nutrition behaviours. Six sections: My profile, My progress, My mates, My groups, My weight and Information centre. General educational materials on PA, nutrition and body weight were provided. Social support included the ability to see and comment on 'mates' progress and complete group challenges. 'ManUp challenge's detailing activity and healthy eating targets were also provided.  Control (n=103): Identical information to internet-based group but in print form and paper log sheets to self-monitor PA, nutrition progress on 'ManUp challenges'.  Mean log-in/week at 9 months =9.22 + 1.47, mean self-monitoring entries at 9 months=22.51 + 3.79, ‘Mate’ feature use n=21, post feature use n=36  46.7% (148/317) retention at 9-months | Nutrition: 19 items adapted from existing instruments. Three dietary outcomes: type of milk consumed, type of bread consumed and an overall index of other dietary behaviours (i.e. dietary score).  Physical activity: Active Australia Questionnaire. Two physical activity outcomes: total minutes of physical activity per week and total number of sessions of physical activity per week. | Nutrition outcomes  *Dietary score*  9-months: Internet vs Control: exp(β) 1.02 (95% CI: 0.98, 1.06), (NS)  *Higher-fiber bread*  9-months: Internet vs Control: exp(β) 1.60 (95% CI: 0.94, 2.71), (NS)  *Low-fat milk*  9-months: Internet vs Control: exp(β) 0.88 (95% CI: 0.52, 1.49), (NS)  Physical activity outcomes  *Minutes of physical activity per week*  9-months: Internet vs Control: exp(β) 1.03 (95% CI: 0.78, 1.36), (NS)  *Sessions of physical activity per week*  9-months: Internet vs Control: exp(β) 0.97 (95% CI: 0.75, 1.25), (NS)  Costs not stated |
| Franko et al [29]  USA  2005-2006 | | 6 university campuses - Northeastern University, College of Charleston, Florida Atlantic University, University of Missouri — St. Louis and Columbia campuses, and Florida International University.  n=606 students randomised  n=476 students completed baseline survey  56.3% (268/476) female  mean age=20.1 years  58.2% (277/476) white  22.9% (109/476) overweight, 12.0% (57/476) obese | Sign up tables in high traffic areas at 6 universities.  Enrolled as a full-time undergraduate student, aged 18-24 years, not attending a formal diet program and not on a special, monitored diet. | Internet I: (n=165): Two sessions of MSB-N (MyStudentBody.com-Nutrition) an internet-based nutrition and physical activity education program (~45mins each). MSB-N included three information links; rate myself assessment; four main  topic pages; and resources.  Internet II (n=164): Two sessions of MSB-N (~45mins each) plus a booster session about 3 weeks later (~45mins).  Control (n=147): Two sessions of an anatomy education website.  Use of internet-based programs (Internet I and Internet II) not described.  69.6% (422/606) retention at 6-months | Nutrition: Food Frequency Questionnaire (FFQ, 16 items) assessed fruit and vegetable intake and percent energy from fat in the last 30 days and single-item measuring number of servings of fruit and vegetables consumed per day.  Physical activity: International Physical Activity Questionnaire (27 items). Vigorous and moderate-intensity activities measured separately in hours, minutes, and days. Each type of activity weighted by its energy requirements, defined in METs, to yield a score in MET minutes. | Nutrition outcomes  *FFQ – daily serves of fruit and vegetables*  Change in mean at 6-months relative to baseline: Internet I: -0.35 vs Control: -0.18 (NS), Internet II: -0.16 vs Control: -0.18 (NS)  *Single-item fruit and vegetable intake*  Change in mean at 6-months relative to baseline: Internet I: 0.13 vs Control: 0.39 (NS), Internet II: 0.40 vs Control: 0.39 (NS)  *FFQ – percent of energy from fat*  Change in mean at 6-months relative to baseline: Internet I: 0.35 vs Control: -0.46 (NS), Internet II: -0.37 vs Control: -0.46 (NS)  Physical activity outcomes  *Total MET minutes/week*  Change in mean at 6-months relative to baseline: Internet I: 112.75 vs Control: -68.00 (NS), Internet II: 133.41 vs Control: -68.00 (NS)  Costs not stated |
| Greene et al [30]  USA  Not stated | | 8 universities – Michigan State University, South Dakota State University, Syracuse University, The Pennsylvania State University, Tuskegee University, University of Rhode Island, University of Maine, and University of Wisconsin  n=1689 students  mean age =19.1 years  62% (1047/1689) female  79% (1334/1689) white  21% (355/1689) overweight and 8% (135/1689) obese | Flyers, table tents in dining halls, newspaper ads and online and class announcements.  Full-time freshmen, sophomores and juniors aged 18-24 years with BMI ≥ 18.5 kg/m^2^ and no health conditions that might interfere with changes in diet and physical activity, not pregnant, lactating or majoring in nutrition or exercise science and completion of baseline assessments. | Internet (n=830): Weekly web-based lesson for 10 weeks and an individualised profile page with data from each physical assessment.  Control (n=859): An individualised profile page with data from each physical assessment.  84% (697/830) completed all 10 lessons and 5.1% (42/830) did not complete any lessons. Average of 7.8 + 4.7 min/lesson for those completing all lessons.  66.7% (1126/1689) retention at 15-months. | Nutrition: Cups per day of fruit and vegetables using the Two-Item Screener and the National Cancer Institute Fruit and Vegetable Screener.  Physical activity: Metabolic equivalent minutes per week (MET-min/wk) of physical activity measured via the International Physical Activity Questionnaire. | *Fruit and vegetable intake (cups/day)*  Two-Item Screener  15-months: Internet: 2.9 + 0.10; Control: 2.3 + 0.19 (Significant)  NCI Fruit and Vegetable Screener  15-months: Internet-based: 3.5 + 0.22; Control: 2.8 + 0.22 (Significant)  *Physical activity (MET-min/wk)*  15-months: Internet: 2627.5 + 108.6; Control: 2357.5 + 102.1 (Significant)  Costs not stated. |
| McConnon et al [31]  UK  2003-2004 | | GP practices in Leeds  n=221  77% (170/221) female  mean age=45.8 years  95% (210/221) white  median BMI=34.4 | Posters and flyers were placed in patient waiting areas in GP practices, inviting potential participants to call the study centre or inform their GP or practice nurse of their interest.  Individuals with a body mass index of 30 or more, aged 18–65 years, able to access the Internet at least once per week and able to read and write in English. | Internet (n=111): The website provided advice, tools and information about dietary and physical activity offered personalised advice and motivational statements. Participants asked to log on to the website at least once a week over trial period.  Control (n=110): Printed information.  53% (59/111) reported using the website at 6-months, 29% (32/111) of those still used the website at 12-months. Mean number of logons over the trial was 15.8 (SD=15.2).  68.8% (152/221) retention at 6-months, 59.3% (131/221) retention at 12 months | Nutrition: Dietary  habits were assessed with a questionnaire used in the UK Women's Cohort Study and included items on methods of cooking, portion size and frequency of consumption of various foods and dieting practices.  Physical activity: Baecke physical activity questionnaire measured work, leisure and sports activity. | Nutrition: Internet vs Control: No significant differences in change in nutrition outcome at 6 or 12 months (NS).  Physical activity: Internet vs Control: No significant differences in change in physical activity outcome at 6 or 12 months (NS).  Total costs: Internet: £992.40; Control: £276.12.  Incremental cost effectiveness: Incremental ratio: £39,248 (£716.28/0.01825) |
| Patrick et al [32]  USA  2004-2006 | | San Diego, California  n=441  100% (441/441) men  mean age =43.9 years  71% (313/441) white non-Hispanic  70.3% (310/441) married/living with partner  33.1% (146/441) postgraduate education  15.6% (69/441) overweight, 84.4%(372/441) obese | Newspaper ads (4 newspapers), radio ads (3 stations), TV news story and flyers in local businesses and organisations such as libraries and medical office waiting rooms.  Men, overweight or obese | Internet (n=224): Initial computerised assessment to tailor recommendations for behavioural targets, weekly web-based learning activities, and individualised feedback on progress. Answers to questions posted on website. Pedometers provided to assist in self-monitoring daily steps to input the data on the web site to assist with goal setting.  Control (n=217): Wait-list control given access to a web site containing general health information about stress, hair loss, worksite injury prevention.  Logged onto internet program an average of 23.4 weeks (SD=16.7) to set weekly goals.  66.0% (291/441) retention at 6-months, 70.1% (309/441) retention at 12-months. | Nutrition: 122-item Fred Hutchison Cancer Research Center Food Frequency Questionnaire. Percent energy from total dietary fat, percent energy from saturated fat, fiber grams per 1,000 kcals/day, and servings for fruits and vegetables per 1,000 kcals/day were examined.  Physical activity: International  Physical Activity Questionnaire (IPAQ) long version. Total walking minutes per day and moderate to vigorous MET minutes per week were examined | Nutrition outcomes  *Percent energy from fat*  6-month: Internet: 32.73 (SE=0.53); Control: 35.81 (SE=0.54); (S)  12-month: Internet: 33.14 (SE=0.53); Control: 36.32 (SE=0.53) (S)  *Percent energy from saturated fat*  6-month: Internet: 10.14 (SE=0.19); Control: 11.48 (SE=0.19) (S)  12-month: Internet: 10.35 (SE=0.19); Control: 11.59 (SE=0.19) (S)  *Fibre grams/1,000 kcal/day*  6-month: Internet: 11.86 (SE=0.27); Control: 9.90 (SE=0.28) (S)  12-month: Internet: 11.34 (SE=0.29); Control: 9.99 (SE=0.29) (S)  *Serves of fruit and vegetables/1,000 kcal/day*  6-month: Internet: 2.11 (SE=0.09); Control: 1.64 (SE=0.09) (S)  12-month: Internet: 2.11 (SE=0.10); Control: 1.73 (SE=0.10) (S)  Physical activity outcomes  *IPAQ total walking mins/day*  6-month: Internet: 84.75 (SE=5.16); Control: 65.31 (SE=5.36) (S)  12-month: Internet: 85.62 (SE=5.38); Control: 69.93 (SE=5.39) (S)  *IPAQ square root MVPA MET (min/week)*  6-month: Internet: 56.91 (SE=2.22); Control: 53.96 (SE=2.29) (NS)  12-month: Internet: 57.95 (SE=2.31); Control: 53.28 (SE=2.32) (NS)  Costs not stated |
| Winett et al [33]  USA  Years not stated | | 14 churches in the south Atlantic region.  n=1,071 church members  67% (718/1071) female  median age =53 years  23% (246/1071) African American  57% (610/1071) overweight or obese | Churches were approached by an introductory letter and phone call to the minister. Church members were recruited via pulpit announcements, flyers, posters, church bulletins and ‘kick off’ luncheons.  All participants were eligible for the nutrition component of the internet program but church members who reported heart or lung disease, asthma, diabetes, kidney/liver disease, or autoimmune diseases estimated low fitness less than 3 metabolic equivalents, bone and joint problems, or life-threatening disease within the last 5 years were required to have medical clearance before participating in the physical activity portion. | 2 of 3 trial arms relevant to this review described.  Internet (n=364; 5 churches): Password protected Guide to Health (GTH) website access. 12 modules successively accessible on a weekly basis. Each module to 5-10 mins to complete and included program interactions and a narrator guide. Content was individualised based on baseline assessment entered by the participant including nutrition and step counts.  Control (n=343; 4 churches): Waitlist  57% (207/364) logged onto internet program, mean modules viewed= 4.60 (SD=5.23), 25% (91/364) viewed all twelve modules.  88.7% (950/1071) retention at 7-months (post-test), 87.3% (935/1071) retention at 16-months (follow-up) | Nutrition. Composite variable from the averaged nutritional content of the Block98 Food Frequency and the family’s food shopping receipts. Values were generated for percent kcal from fat, fiber g/1,000 kcal, and servings of fruit and vegetables/1,000 kcal.  Physical activity. Wore a pedometer to track steps on a daily log for 7 consecutive days without resetting the pedometer. Mean daily step counts computed for the number of days steps were recorded. | Nutrition outcomes  *Fibre g/1,000kcal (mean)*  7-months (post-test): Internet:1.62 (SE=0.19); Control: 0.71 (SE=0.19) (S)  16-months (follow-up): Internet:1.64 (SE=0.23); Control: 0.91 (SE=0.22) (S)  *Fruit and Vegetables/1000kcal* *(mean)*  7-months (post-test): Internet:0.80 (SE=0.10); Control: 0.30 (SE=0.10) (S)  16-months (follow-up): Internet:0.59 (SE=0.10); Control: 0.24 (SE=0.10) (S)  *Percent kcal from fat (mean)*  7-months (post-test): Internet: -0.14 (SE=0.47); Control: 0.66 (SE=0.46) (NS)  16-months (follow-up): Internet:0.76 (SE=0.51); Control: 1.11 (SE=0.50) (NS)  Physical activity outcome  *Steps/day (mean)*  7-months (post-test): Internet: 1408.57 (SE=363.03); Control: 398.50 (SE=386.34) (NS)  16-months (follow-up): Internet: 704.70 (SE=277.13); Control: -38.49 (SE=270.12) (NS)  Costs not stated |

S= Significant, NS=not significant
