# Supplementary material for: The Long-Term Effectiveness of Internet-Based Interventions on Multiple Health Risk Behaviors: Systematic Review and Robust Variance Estimation Meta-analysis
Source: J Med Internet Res. 2021 Dec 21;23(12):e23513. doi: 10.2196/23513 (PMC8734928; doi:10.2196/23513)
Supplement: Multimedia Appendix 2 [file jmir_v23i12e23513_app2.docx]

**Multimedia Appendix 2: Characteristics of studies examining the effectiveness of an internet-based intervention on smoking, nutrition and physical activity**

| **Authors**  **Country Years data collected** | **Setting**  **Sample characteristics (sample size, demographics)** | **Recruitment method**  **Eligibility criteria** | **Treatment conditions (relevant arms)**  **Internet-based intervention received**  **Retention at follow-up** | **Measures of health behaviours** | **Health behaviours outcomes** **Costs** |
| --- | --- | --- | --- | --- | --- |
| Hughes et al [34]  USA  2006-2008 | A university - University of Illinois at Chicago  n=423 staff  82% (347/423) female  mean age=51 years  45% (190/423) African American  57% (241/423) college degree  mean length of employment at university=12.6 years  32% (135/423) overweight, 46% (195/423) obese | Announcements on staff listservs, e-mails, staffed recruitment tables at events in highly trafficked buildings and flyers posted throughout university.  Support and academic university staff aged ≥ 40 years with reliable access to the internet at home or work. | 2 of 3 trial arms relevant to this review described.  Internet (RealAge) (n=135): Website that included a test and generated individual risk profiles and indicated areas that could be worked on to improve health. Used website to select behaviours to work on and create plans to meet behavioural goals.  Control (n=138): Printed health-promotion materials.  59% (79/135) completed all sections of test to gain unlimited access to the website and 53% (72/135) clicked at least one link to obtain additional information.  85.1% (360/423) retention at 6 months and 86.8% (367/423) at 12 months | Smoking: Smoking cessation defined as a minimum of 6 months of total abstinence from tobacco use at 6 months and 12 months.  Nutrition: The National Cancer Institute’s Percentage Energy from Fat Screener questionnaire assessed usual daily intake of fat and the National Cancer Institute All-Day Fruit and Vegetable Screener questionnaire examined fruit and vegetable intake during a typical day.  Physical activity. The 7-item Behavioral Risk Factor Surveillance System scale assessed vigorous and moderate physical activity over a typical week. The Rapid Assessment of Physical Activity (RAPA) assessed exercise participation. | Smoking cessation 12-months: Internet: 25% (4/16); Control: 14.3% (3/21) (NS)  Nutrition outcomes Percentage energy from fat 6-months: Internet vs Control: coefficient= -0.729 (NS)  12-months: Internet vs Control: coefficient= -1.543 (NS) Fruit and vegetable consumption 6-months: Internet vs Control: coefficient= -0.110 (NS)  12-months: Internet vs Control: coefficient= 1.541 (NS)  Physical activity outcomes Minutes of moderate activity 6-months: Internet vs Control: coefficient= 0.337 (NS)  12-months: Internet vs Control: coefficient= 0.108 (NS) Minutes of vigorous activity 6-months: Internet vs Control: coefficient= 0.419 (NS)  12-months: Internet vs Control: coefficient= 0.283 (NS)  *Rapid Assessment of Physical Activity*  6-months: Internet vs Control: coefficient= -0.395 (NS)  12-months: Internet vs Control: coefficient= -0.047 (NS)  Costs not stated |

NS=not significant
