# Supplementary material for: The Long-Term Effectiveness of Internet-Based Interventions on Multiple Health Risk Behaviors: Systematic Review and Robust Variance Estimation Meta-analysis
Source: J Med Internet Res. 2021 Dec 21;23(12):e23513. doi: 10.2196/23513 (PMC8734928; doi:10.2196/23513)
Supplement: Multimedia Appendix 3 [file jmir_v23i12e23513_app3.docx]

**Multimedia Appendix 3: Characteristics of studies examining the effectiveness of an internet-based intervention on SNAP**

| **Authors**  **Country Years data collected** | **Setting**  **Sample characteristics (sample size, demographics)** | | **Recruitment method**  **Eligibility criteria** | **Treatment conditions (relevant arms)**  **Internet-based intervention received**  **Retention at follow-up** | **Measures of health behaviours** | **Health behaviours outcomes**  **Costs** |
| --- | --- | --- | --- | --- | --- | --- |
| Cameron et al [35]  UK  2013– 2014 | A university - University of Sheffield  n=2621 undergraduate students  55.4% (1447/2614)* female  mean age=18.8 years  77.0% (1937/2514)* white  *denominators vary due to missing data | | All incoming undergraduate students were sent an e-mail inviting them to take part in the study 3 weeks prior to starting university.  Incoming undergraduate students | Internet (n=1346): U@Uni:LifeGuide website access to complete a profile page containing self-affirmation manipulation and then directed to complete four short modules on each of the four health behaviours that contained theory-based messages and planning exercises. After completing all four modules, had access to the full website with further health messages and links on each of the health behaviours and a planner to form implementation intentions.  Control (n= 1275): no intervention  85% (1149/1346) completed the self-affirmation task, 72% (973/1346) viewed a health message for at least one behaviour, 50% (672/1346) for at least two behaviours, 48% (640/1346) for at least three behaviours, and 47% (630/1346) for four behaviours. 41% (554/1346) formed an implementation intention for at least one behaviour, 36% (479/1346) for at least two behaviours, 33% (439/1346) for at least three behaviours, and 29% (395/1346) for four behaviours.  41.2% (1079/2621) retention at 6-months. | Primary outcomes  Smoking: Current tobacco smoking using items based on the Health Survey for England.  Nutrition: Fruit and vegetable intake (portions per day) measured using a two-item dietary questionnaire.  Alcohol: A 7-day recall drinking diary, where reported the amount of alcohol (units) consumed on each of the previous 7 days.  Physical activity: Short Form of International Physical Activity Questionnaire assessed how many times, and for how long, engaged in vigorous exercise, moderate exercise and walking in the past 7 days. Responses converted into METs (metabolic equivalent of task). | *Current smoking*  6-months: Internet: 11.18% (75/671); Control: 14.05% (110/783) (NS)  *Fruit and vegetable intake (mean portions per day)*  6-months: Internet: 4.11 (SD=1.84); Control: 3.89 (SD=1.97) (NS)  *Alcohol in last 7 days (mean units)*  6-months: Internet: 10.42 (SD=10.86); Control: 11.03 (SD=10.91) (NS)  *Physical activity in last 7 days (mean METs)*  6-months: Internet: 3627.94 (SD=2578.97); Control: 3613.27 (SD= 2578.07) (NS)  Costs not stated |
| Epton et al [36]  UK  2012 – 2013 | A university - University of Sheffield  n=1445 undergraduate students  58.4% (844/1445) female  mean age 18.9 years  73.2% (1052/1438)* white  *denominator varies due to missing data | | All incoming undergraduate students were sent an e-mail inviting them to take part in the study 2 weeks prior to starting university.  Incoming undergraduate students | Internet (n=736): U@Uni website access to complete a profile page containing self-affirmation manipulation. The website contained online resources which included theory-based messages relevant to each of the four targeted health behaviours, a planner containing instructions to form implementation intentions and access to more detailed information.  Control (n= 709): no intervention  At 6 months, 52% (383/736) had completed the self-affirmation task, 35% (259/736) had accessed the health messages, and 1%(8/736) had made a plan.  63.2% (913/1445) retention at 6-months. | Primary outcomes  Smoking: Current tobacco smoking using items based on the Health Survey for England (HSE).  Nutrition: Fruit and vegetable intake (portions per day) measured with items based on the HSE.  Alcohol: Units of alcohol per week using items from the General Lifestyle Survey.  Physical activity: Short Form of International Physical Activity Questionnaire (IPAQ-SF) assessed how many times, and for how long, engaged in vigorous exercise, moderate exercise and walking in the past 7 days. Responses converted into METs (metabolic equivalent of task). | *Current smoking*  6-months: Internet: 8.7% (47/540); Control: 13.0% (72/553) smokers (S)  *Fruit and vegetable intake (mean portions per day)*  6-months: Internet: 5.61 (SD=4.89), Control :5.72 (SD=4.98) (NS)  *Alcohol in last 7 days (mean units)*  6-months: Internet 13.01 (SD=19.75); Control 13.41 (SD=19.65) (NS)  *Physical activity in last 7 days (mean METs)*  6-months: Internet: 3350.52 (SD=5144.16); Control: 3316.10 (SD=5143.79) (NS)  Costs not stated |
| Tapper et al [37]  UK  2012 | | UK community    n=100  82% (82/100) female  mean age=39 years  93% (93/100) white  63% (63/100) degree level educated  mean BMI=27.68 kg/m^2^ | Online and print advertisements in the local community including; posters and flyers in local shops and community facilities, advertisements on social media sites, email networks and in local newspapers.  Aged 18 years or older, able to attend laboratory appointments and complete weekly online sessions, were not pregnant, were not out of the country for 3 weeks during study period, did not have another household member already participating and did not participate in another related study. | Internet (n=50): HealthyValues Healthy Eating programme promoting behaviour change alongside psychological principles, with phases targeting motivation (dietary feedback and advice, analysing reasons for health values, thinking about health-related desires, and concerns), volition (implementation intentions with mental contrasting), and maintenance (reviewing tasks, weekly tips). Weekly program tasks for 24 weeks.  Control (n=50): no intervention  95% (95/100) retention at 6 months. | Smoking: Cigarette smoking status and number of cigarettes per week.  Nutrition: The Block Fat/Sugar/Fruit/Vegetable screener, a 55-item food frequency questionnaire (FFQ) measured: (1) saturated fat, (2) added sugar, and (3) fruit and vegetables. Frequency and quantity of intake examined.  Alcohol: 4-item questionnaire designed to capture episodes of binge drinking and typical drinking behaviours. Frequency of consumption assessed and number of units consumed for both usual consumption and for days when consumed larger-than-usual quantities.  Physical activity: Short version of the International Physical Activity Questionnaire (IPAQ). Assessed number of days and duration engaged in vigorous activity, moderate activity and walking during previous week. Converted into total number of metabolic equivalent of task (MET) units expended per day. | Smokers (per-protocol analyses):  6-months: Internet: n=3; Control: n=4 (NS)  Nutrition outcomes (intention-to-treat)  *Saturated fat (mean grams)*  6-months: Internet: 15.7 (SD=9.9); Control: 15.9 (SD=6.6) (NS)  *Added sugar (mean grams)*  6-months: Internet: 30.5 (SD=37.0); Control: 38.5 (SD=37.6) (NS)  *Fruit and vegetables (mean cups)*  6-months: Internet: 3.9 (SD=1.6); Control: 3.3 (SD=1.5) (NS)  Alcohol outcomes (per-protocol analyses):  *Alcohol (units per week)*  6-months: Internet: 6.7 (SD=7.3); Control: 7.2 (SD=7.5) (NS)  *Binge drinking*  6-months: Internet: n=17; Control: n=20 (NS)  *Physical activity (METs per week)* (per-protocol analyses):  24 weeks: Internet: 2350 (SD=2344); Control: 2985 (SD=3525) (NS)  Costs not stated |

## S= Significant, NS=not significant
